# Clinical carbapenem-resistant *Klebsiella pneumoniae* isolates simultaneously harboring *bla*_NDM-1_, *bla*_OXA_ types and qnrS genes from the Kingdom of Bahrain: Resistance profile and genetic environment

**DOI:** 10.3389/fcimb.2022.1033305

**Published:** 2022-10-11

**Authors:** Mohammad Shahid, Nayeem Ahmad, Nermin Kamal Saeed, Mohd Shadab, Ronni Mol Joji, Ali Al-Mahmeed, Khalid M. Bindayna, Khaled Saeed Tabbara, Fazal K. Dar

**Affiliations:** ^1^ Department of Microbiology, Immunology, and Infectious Diseases, College of Medicine & Medical Sciences, Arabian Gulf University, Manama, Bahrain; ^2^ Department of Pathology, Microbiology Section, Salmaniya Medical Complex, Manama, Bahrain

**Keywords:** carbapenem-resistant *klebsiella pneumoniae*, antibiotics, integron, plasmids, hospital, polymerase chain reaction

## Abstract

The prevalence of Carbapenem-resistant *Klebsiella pneumoniae* (CRKP) is currently increasing worldwide, prompting WHO to classify it as an urgent public health threat. CRKP is considered a difficult to treat organism owing to limited therapeutic options. In this study, a total of 24 CRKP clinical isolates were randomly collected from Salmaniya Medical Complex, Bahrain. Bacterial identification and antibiotic susceptibility testing were performed, on MALDI-TOF and VITEK-2 compact, respectively. The isolates were screened for carbapenem resistance markers (*bla*_NDM,_ *bla*_OXA-23,_ *bla*_OXA-48_ and *bla*_OXA-51_) and plasmid-mediated quinolone resistance genes (*qnrA, qnrB*, and *qnrS*) by monoplex PCR. On the other hand, only colistin-resistant isolates (n=12) were screened for MCR-1, MCR-2 and MCR-3 genes by monoplex PCR. Moreover, the Genetic environment of *bla*_NDM_, integrons analysis, and molecular characterization of plasmids was also performed. Antibiotic susceptibility revealed that all the isolates (100%) were resistant to ceftolozane/tazobactam, piperacillin/tazobactam, 96% resistant to ceftazidime, trimethoprim/sulfamethoxazole, 92% resistant to meropenem, gentamicin and cefepime, 88% resistant to ciprofloxacin, imipenem, and 37% resistant to amikacin. Ceftazidime/avibactam showed the least resistance (12%). 75% (n=12/16) were resistant to colistin and 44% (n=7/16) showed intermediate susceptibility to tigecycline. The detection of resistant determinants showed that the majority (95.8%) of CRKP harbored *bla*_NDM-1_, followed by *bla*_OXA-48_ (91.6%) *bla*_OXA-51_ (45.8%), and *bla*_OXA-23_ (41.6%). Sequencing of the *bla*_NDM_ amplicons revealed the presence of *bla*_NDM-1_. Alarmingly, 100% of isolates showed the presence of *qnrS*. These predominant genes were distributed in various combinations wherein the majority were *bla*_NDM-1_ + *bla*_OXA-51_+ *qnrS* + *bla*_OXA-48_ (n =10, 41.7%), *bla*_NDM-1_ + *bla*_OXA-23_+ *qnrS* + *bla*_OXA-48_ (n=8, 33.3%), among others. In conclusion, the resistance rate to most antibiotics is very high in our region, including colistin and tigecycline, and the genetic environment of CRKP is complex with the carriage of multiple resistance markers. Resistance to ceftazidime/avibactam is uncommon and hence can be used as a valuable option for empirical therapy. Molecular data on resistance markers and the genetic environment of CRKP is lacking from this geographical region; this would be the first report addressing the subject matter. Surveillance and strict infection control strategies should be reinforced in clinical settings to curb the emergence and spread of such isolates.

## Introduction

The emergence and increase in multidrug-resistant bacteria have become a global health threat (Carlet et al., 2012). The World Health Organization identified antibiotic resistance as one of the top 10 most important global health threats prior to the COVID-19 pandemic. However, there are growing worries that the COVID-19 pandemic would hinder future efforts to combat antibiotic resistance (Choudhury et al., 2022). According to the Centers for Disease Control and Prevention, antibiotic resistance in the US increased by 15% overall between 2019 and the period immediately following the pandemic’s peak in 2020 (Tanne, 2022). Multidrug-resistant Enterobacterales in particular are a leading cause of healthcare-associated infections, which are linked to increased morbidity and death, as well as rising medical expenditures (Friedman et al., 2016). Globally the prevalence of carbapenem-producing Enterobacterales varies significantly (Logan and Weinstein, 2017). Carbapenem-resistant *Klebsiella pneumoniae* (CRKP), a member of the carbapenem-resistant Enterobacterales (CRE) family, is an emerging cause of healthcare-associated infections worldwide (Band et al., 2018). Worst of all, CRKP can pass on its resistance to other bacteria *via* horizontal gene transfer, which can include bacterial conjugation, resulting in drug resistance. Two primary mechanisms induce carbapenem resistance. First, CRKP can produce β-lactamases that can hydrolyze cephalosporins, or ESBLs combined with reduced cell wall permeability. Second, the formation of β-lactamases, which can hydrolyze most β-lactams, including carbapenems. Conferring to the Ambler classification, it belongs to class A (KPC, SME, IMI, GES, NMC), class B (NDM, IMP, and VIM family), and class D (OXA-48 like) (Pitout et al., 2015). Because of the capability of hydrolyzing all β-lactam antibiotics except aztreonam, class B enzymes are the most clinically relevant carbapenemases (Elshamy and Aboshanab, 2020).

Acquired Metallo-β-lactamases (MBL) including IMP, VIM, and NDM carbapenemases in Enterobacterales are reported in the literature (Bush and Bradford, 2020). The NDM-1 gene was originally discovered in a Swedish patient (Indian origin) in New Delhi in 2008 (Yong et al., 2009). Since then, NDM-1 producers have been reported in the United Kingdom, Sweden, France, Germany, China, Belgium, Japan, Austria, Australia, Norway, Canada, and the Netherlands (Khan et al., 2017). Since the first *bla*_NDM-1_ positive *K. pneumoniae* isolates were discovered in Nanchang, China in 2013, the disease has spread swiftly throughout the country. In Shanghai, Hunan, Yunnan, and other places, outbreaks of NDM-1 generating *K. pneumoniae* have been recorded (Zhu et al., 2016). NDM cases are also reported from the Indian sub-continent (Ahmad et al., 2018) and the Middle East (Al-Zahrani and Alsiri, 2018).

Class-D β-lactamase, also known as OXA-type enzymes or oxacillinases, is a group of about 400 genetically distinct enzymes (Khan et al., 2022). Only a limited fraction of the class-D, OXA family functions as a carbapenemase. The common OXA-48, OXA-23, OXA-40, as well as its variations OXA-232, OXA-162, and OXA-181, are among them (Mairi et al., 2018). OXA-48-like enzyme is among the most common carbapenemases in Enterobacterales. Despite the low β-lactamase activity, this enzyme hydrolyzes penicillin and is uninhibited by β-lactamase inhibitors (Pfeifer et al., 2012). Since the discovery of OXA-48 carbapenemase in Turkey in 2001, these strains have been implicated in numerous nosocomial outbreaks around the world (van Duin and Doi, 2017), including Middle East (Shibl et al., 2013), the Mediterranean region (van Duin and Doi, 2017) and European countries (European Centre for Disease Prevention and Control, 2019). OXA-48 *K. pneumoniae* is endemic to North Africa and the Middle East, while OXA-181 *K. pneumoniae* is found in India. However, OXA-181 nosocomial epidemics have been reported in Sub-Saharan Africa (Pitout et al., 2019).

Although few researches on CRKP isolates have been undertaken in the Arabian Peninsula, nearly all Gulf Cooperation Council (GCC) countries share similar ESBLs and carbapenemases-producing Enterobacterales, the majority of which were isolated from nosocomial infections (Al-Zahrani and Alsiri, 2018). Furthermore, a review article on gram-negative bacilli producing β-lactamases from GCC states revealed that the most prevalent and widespread β-lactamases genes are NDM-1, and OXA-48 (Zowawi et al., 2013). Owing to the real difficulty of treating CRKP, epidemiological analysis of carbapenemases coding genes in circulating strains is critical for designing strategies to decrease infection outbreaks and creating novel therapeutic techniques (Lombardi et al., 2015). This geographical region lacks exhaustive molecular data on resistance markers and the genetic environment of CRKP. Therefore, this study aimed to analyze the antibiotic resistance profile and genetic environment among clinical CRKP isolates from the Kingdom of Bahrain.

## Material and methods

### Ethics statement

The protocol of this study was reviewed and approved by the Research Ethics Committee, Arabian Gulf University (AGU) (E012-PI-11/19) and the Ministry of Health (AURS/305/2020).

### Bacterial isolates and hospital setting

From December 2020 to June 2021, twenty-four non-duplicate CRKP clinical isolates were included in this study. These were isolated from the blood, urine and endotracheal aspirate, of the patients, admitted to tertiary care hospital (Salmaniya Medical Complex), Kingdom of Bahrain.

### Bacterial identification and antimicrobial susceptibility testing

Bacterial species-level identification was confirmed by using a mass spectrometry system (MALDI-TOF Bruker Daltonik GmbH, Bremen, Germany) and antibiotic susceptibility testing of isolates was performed with automated microbiological systems (VITEK-2 compact bioMerieux, Marcy L, Etoile, France). Only the isolates that were identified as *K. pneumoniae* resistant to carbapenems were included for further molecular analysis.

### Amplification of antibiotic-resistant genes by Polymerase chain reaction

For antibiotic-resistant gene detection, whole-cell DNA of strains was prepared from CRKP pure culture. Suspension of each colony was done in 100 µl of nuclease-free sterilized water and incubated at 94°C for 20 min followed by centrifugation at 6,000*g* at 4°C for 20min. The supernatant was used as a template to perform PCR on a GeneAmp PCR System 9700 (Applied Biosystems, Foster City, CA) using the specific primers as mentioned in **Table S1** for the detection of carbapenem-resistance genes (*bla*_KPC,_ *bla*_NDM,_ *bla*_OXA-23,_ *bla*_OXA-48,_ *bla*_OXA-51_), plasmid-mediated quinolone resistance genes (*qnrA, qnrB, qnrS*) and colistin-resistance plasmid-mediated genes (MCR-1, MCR-2, MCR-3).

### DNA sequencing

At Genoscreen Lab, PCR-generated fragments were sequenced (Campus Institut Pasteur de, France). Using the Clustal omega tool (https://www.ebi.ac.uk/Tools/msa/clustalo/), the derived protein sequence was aligned with *bla*_NDM_ variants to verify the amino acid substitution in the query sequence for known variants. Additionally, online BLAST software (http://www.ncbi.nlm.nih.gov/BLAST/) was used to analyze the similarities between the amplified nucleotide sequence and the deduced protein sequences and was confirmed as *bla*_NDM-1._ Under the following accession numbers, these sequences have been added to the GenBank nucleotide database: ON506904, ON506905, ON506906, ON506907, ON506908, ON506909, ON506910, ON506911, ON506912, ON506913, ON506914, ON506915, ON506916, ON506917, ON506918, ON493161, ON493162, ON493163, ON755345 and ON755346 accessible at the National Center of Biotechnology Information website (http://www.ncbi.nlm.nih.gov).

### Isolation and separation of plasmid DNA

Plasmid DNA was isolated from CRKP clinical isolates using the Qiagen Plasmid Mini Kit following the manufacturer’s instructions, which included steps 1 and 2 of clearing a bacterial lysate, adsorbing DNA onto the QIAprep membrane, and steps 3 washing and elution of plasmid. The isolated Plasmid DNA samples were then electrophoresed in 0.9% agarose gel stained with ethidium bromide (0.5 μg/ml) and visualized under an ultraviolet Gel documentation azure Biosystem C-200.

### Genetic environment analysis

The genes present upstream and downstream of *bla*_NDM,_ was identified by the Genetic Environment analysis as described previously (Poirel et al., 2011b).

### Integron analysis

Detection and characterization of class 1, 2 and 3 integrons in CRKP isolates were investigated by amplification of integrase genes including *intI1*, *intI2*, and *intI3* by using specific primers mentioned in **Table S1**.

## Results

### Distribution and antibiotic resistance pattern of the isolates

**Figure 1:**
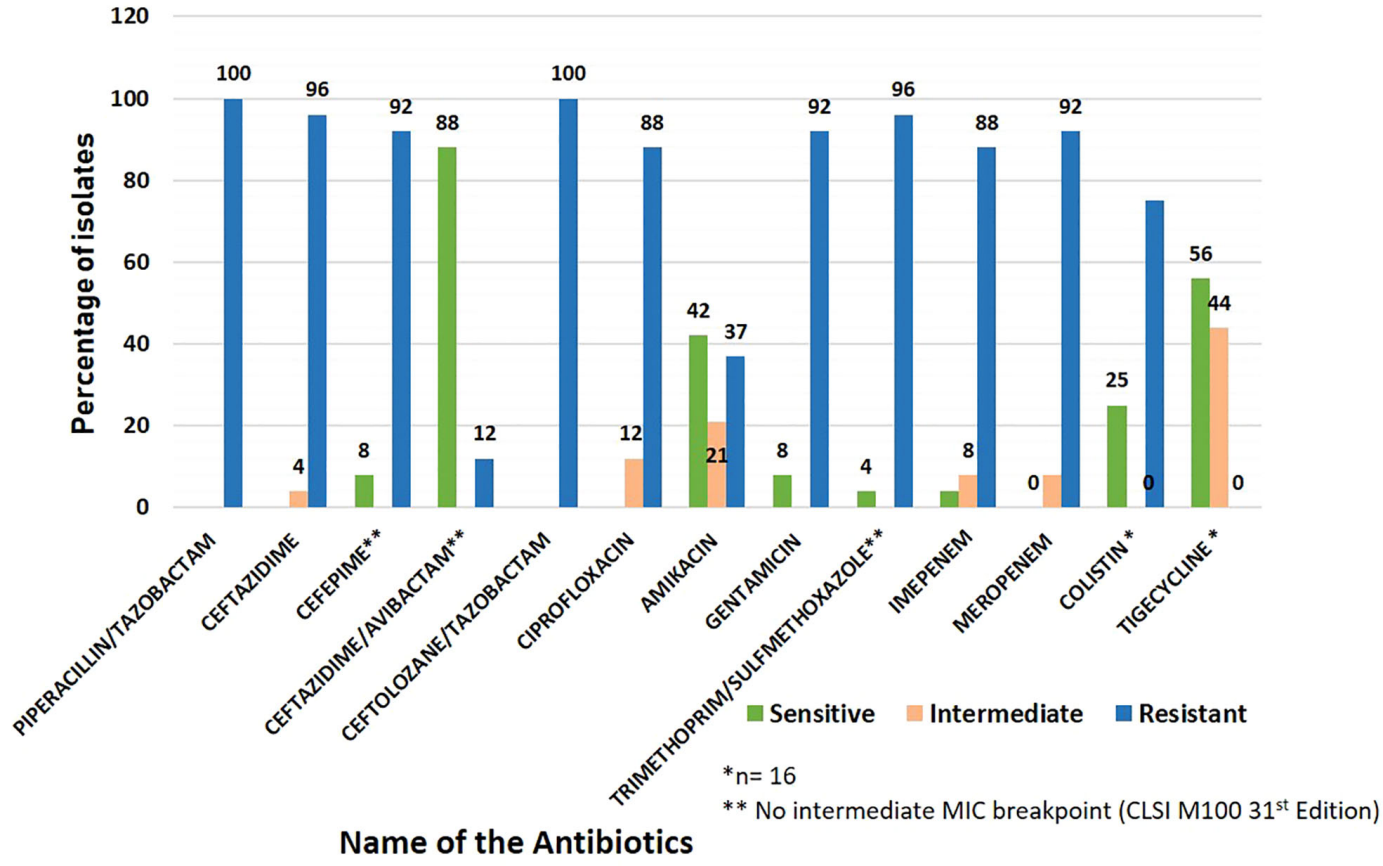
Antibiotic susceptibility pattern in carbapenem-resistant Klebsiella pneumoniae (CRKP) clinical isolates included in the study.

Blood provided the majority of the CRKP clinical isolates (n=17, 70.8%), followed by urine (n=5, 20.8%) and endotracheal aspirate (n=2, 8.3%). Antibiotic susceptibility revealed that all the isolates (n=24, 100%) were resistant to ceftolozane/tazobactam and piperacillin/tazobactam, 96% (n=23) resistant to ceftazidime and trimethoprim/sulfamethoxazole, 92% (n=22) showed resistance to meropenem, gentamicin and cefepime, 88% (n=21) resistant to ciprofloxacin, imipenem, and 37% (n=9) resistant to amikacin. Ceftazidime/avibactam showed the least resistance (n=3, 12%). Among the 16 isolates tested for colistin and tigecycline resistance, 75% (n=12) were resistant to colistin and 44% (n=7) showed intermediate susceptibility to tigecycline (**Figure 1**).

### Minimum inhibitory concentrations

The CRKP clinical isolates showed high Minimum inhibitory concentrations (MICs) against frequently used antibiotics in clinical settings vis-à-vis Amikacin, Cefepime, Ceftazidime, Ceftolozane/tazobactam, Ciprofloxacin, Gentamicin, Meropenem, Piperacillin/tazobactam, and Trimethoprim/sulfamethoxazole as shown in **Table 1**.

**Table 1:** Minimum Inhibitory Concentrations (MICs) distribution of respective antibiotics in carbapenem-resistant *Klebsiella pneumoniae* (CRKP) clinical isolates tested in the study.

S.No	Isolates Id	MIC (µg ml^-1^)										Carbapenem resistance genes	Quinolone resistance genes
		AK	CPM	CAZ	CAZ/AVI	C/T	CIP	GEN	MEP	PIP/TAZ	TMP/SULFA		
1.	MIID-C1	>32	>16	>16	4/4	>8/4	>2	>8	32	>64/4	>2/38	*bla*_NDM-1,_ *bla*_OXA-23,_ *bla*_OXA-48_	*qnrS*
2.	MIID-C2	32	>16	>32	1	>16	>2	>=16	2	>64	>160	*bla*_NDM-1,_ *bla*_OXA-23_	*qnrS*
3.	MIID-C3	32	>16	>32	1	>16	>2	>8	>8	>64	>160	*bla*_NDM-1,_ *bla*_OXA-23,_ *bla*_OXA-48_	*qnrS*
4.	MIID-C4	32	>16	>32	4	>16	>2	>8	>8	>64	>160	*bla*_NDM-1,_ *bla*_OXA-23,_ *bla*_OXA-48_	*qnrS*
5.	MIID-C5	>32	>16	>16	4/4	>8/4	>2	>8	32	>64/4	>2/38	*bla*_NDM-1,_ *bla*_OXA-23,_ *bla*_OXA-48_	*qnrS*
6.	MIID-C6	>32	>16	>16	<=0.25/4	>8/4	>2	>8	32	>64/4	>2/38	*bla*_NDM-1,_ *bla*_OXA-23,_ *bla*_OXA-48_	*qnrS*
7.	MIID-C7	>32	>16	>16	4/4	>8/4	>2	>8	32	>64/4	>2/38	*bla*_NDM-1,_ *bla*_OXA-23,_ *bla*_OXA-48_	*qnrS*
8.	MIID-C8	>32	>16	>16	2/4	>8/4	>2	>8	32	>64/4	>2/38	*bla*_NDM-1,_ *bla*_OXA-23,_ *bla*_OXA-48_	*qnrS*
9.	MIID-C9	>32	>16	>16	2/4	>8/4	>2	>8	32	>64/4	>2/38	*bla*_NDM-1,_ *bla*_OXA-48_	*qnrS*
10.	MIID-C11	>32	>16	>16	2/4	>8/4	>2	>8	32	>64/4	>2/38	*bla*_OXA-51,_ *bla*_OXA-48_	*qnrS*
11.	MIID-C12	>32	>16	>16	2/4	>8/4	>2	>8	>32	>64/4	>2/38	*bla*_NDM-1,_ *bla*_OXA-23,_ *bla*_OXA-48_	*qnrS*
12.	MIID-C13	>32	>16	>16	0.5/4	>8/4	>2	>8	>32	>64/4	>2/38	*bla*_NDM-1,_ *bla*_OXA-48_ *bla*_OXA-51_	*qnrS*
13.	MIID-C15	<=4	>16	>16	0.5/4	>8/4	>2	>8	32	>64/4	>2/38	*bla*_NDM-1,_ *bla*_OXA-48_ *bla*_OXA-51_	*qnrS*
14.	MIID-C16	8	>16	>16	>8/4	>8/4	2	>8	>32	>64/4	>2/38	*bla*_NDM-1,_ *bla*_OXA-23_	*qnrS*
15.	MIID-C17	<=4	>16	>16	0.5/4	>8/4	>2	>8	32	>64/4	>2/3	*bla*_NDM-1,_ *bla*_OXA-48_ *bla*_OXA-51_	*qnrS*
16.	MIID-C18	<=4	>16	>16	0.5/4	>8/4	2	>8	32	>64/4	>2/38	*bla*_NDM-1,_ *bla*_OXA-48_ *bla*_OXA-51_	*qnrS*
17.	MIID-C19	<=4	>16	>16	1/4	>8/4	>2	>8	32	>64/4	>2/38	*bla*_NDM-1,_ *bla*_OXA-48_ *bla*_OXA-51_	*qnrS*
18.	MIID-C20	<=4	>16	>16	1/4	>8/4	>2	>8	32	>64/4	>2/38	*bla*_NDM-1,_ *bla*_OXA-48_ *bla*_OXA-51_	*qnrS*
19.	MIID-C21	8	>16	>16	1/4	>8/4	>2	>8	32	>64/4	>2/38	*bla*_NDM-1,_ *bla*_OXA-48_ *bla*_OXA-51_	*qnrS*
20.	MIID-C22	32	>16	>32	>=16/4	>16	>2	>8	>8	>64/4	>160	*bla*_NDM-1,_ *bla*_OXA-48_ *bla*_OXA-51_	*qnrS*
21.	MIID-C23	2	1	32	4	>16	0.5	<=1	4	>64	<=20	*bla*_NDM-1,_ *bla*_OXA-48_	*qnrS*
22.	MIID-C24	<=1	2	8	1	8	0.5	<=1	2	64	>160	*bla*_NDM-1,_ *bla*_OXA-48_ *bla*_OXA-51_	*qnrS*
23.	MIID-C25	16	>16	>32	>=16/4	>16	>2	>8	>8	>64	>160	*bla*_NDM-1,_ *bla*_OXA-48_ *, bla*_OXA-51_	*qnrS*
24.	MIID-C26	32	>16	>32	1	>16	>2	>8	8	>64	>160	*bla*_NDM-1,_ *bla*_OXA-48,_	*qnrS*

AK, Amikacin; CPM, Cefepime; CAZ, Ceftazidime; CAZ/AVI, Ceftazidime/Avibactam; C/T, Ceftolozane/Tazobactam; CIP, Ciprofloxacin; GEN, Gentamicin; MEP, Meropenem; PIP/TAZ, Piperacillin-tazobactam; TMP/SULFA, Trimethoprim/Sulfamethoxazole.

### Antimicrobial resistance genes detection

All the isolates subjected to PCR assays to detect resistant determinants to β-lactam antibiotics (carbapenems and cephalosporins) showed that the majority of CRKP harbored *bla*_NDM-1_ (95.8%; 23/24), followed by *bla*_OXA-48_ (91.6%; 22/24) *bla*_OXA-51_ (45.8%; 11/24), and *bla*_OXA-23_ (41.6%; 10/24). None of our isolates showed the presence of *bla*_KPC_ gene. Sequencing of the *bla*_NDM_ amplicons revealed the confirmation of *bla*_NDM-1_. Alarmingly, 100% of isolates showed the presence of *qnrS*. These predominant genes were distributed in various combinations: *bla*_NDM-1_ + *bla*_OXA-51_+*qnrS*_+_ *bla*_OXA-48_ (n =10, 41.7%), *bla*_NDM-1_ + *bla*_OXA-23_+ *qnrS*_+_ *bla*_OXA-48_ (n=8, 33.3%), *bla*_NDM-1_ + *qnrS*+ *bla*_OXA-48_ (n=3, 12.5%), *bla*_NDM-1_+ *bla*_OXA-23_ + *qnrS* (n=2, 4.2%), *bla*_OXA-51_ + *qnrS*+ *bla*_OXA-48_ (n=1, 4.2%). None of the colistin-resistant isolates showed the presence of plasmid-mediated MCR genes that we screened. Here probably other resistance mechanisms are likely to have a role in colistin resistance. No correlation was found between various gene combinations and MIC values of carbapenem.

### Plasmid profiling

**Figure 2:**
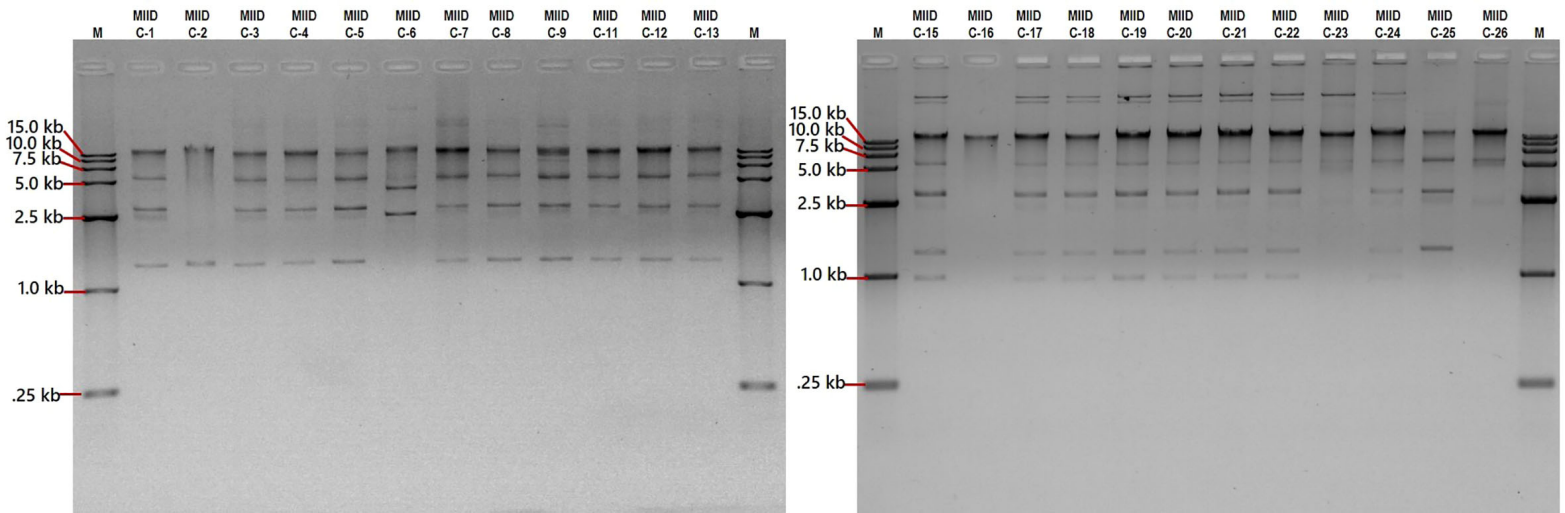
Separation of plasmid DNA molecular weight on 0.9% agarose gel stained with ethidium bromide. Lanes M, DNA marker 15.0 kb (cat. no. 239135), lanes MIID-C1 to MIID-26 CRKP clinical isolates carrying different sizes of plasmids.

Plasmid profiling was carried out on each of the CRKP isolates to gather an insight on similarity (or dissimilarity) in types/number of plasmids carried by these isolates. The number of plasmids carried in these isolates were as follows: Eight isolates (MIID-C9, MIID-C15, MIID-C17, MIID-C18, MIID-C19, MIID-C20, MIID-C21 and MIID-C22) carried seven plasmids; two isolates (MIID-5 and MIID-8) carried six plasmids; eight isolates (MIID-C1, MIID-C3, MIID-C4, MIID-C6, MIID-C7, MIID-C11, MIID-C12 and MIID-C13) carried five plasmids; two isolates (MIID-24 and MIID-25) carried four plasmids; three isolates (MIID-2, MIID-23 and MIID-26) carried two plasmids; and one isolate (MIID-16) carried single plasmid, respectively. The plasmid sizes ranged approximately from 1.0 to 18.1 kb, with the most prevalent plasmid being approximately 1.5 kb in size and found in all of the CRKP clinical isolates (**Table 2**; **Figure 2**).

**Table 2:** Genetic characterization of carbapenem-resistant *Klebsiella pneumoniae* (CRKP) clinical isolates.

S.No	Isolate Id	Sample source	Organism name	GenBank Accession No.	Carbapenem resistance-genes	Quinolone resistance genes	Plasmid-Molecular size in kb	Number of Plasmids	Integron	Genetic environment of *bla*_NDM_	
										IS*Aba125*	*ble*_MBL_
1.	MIID-C1	Urine	*K. pneumoniae*	ON506904	*bla*_NDM-1,_ *bla*_OXA-23,_ *bla*_OXA-48_	*qnrS*	1.5, 2.5, 2.9, 5.8, 15.2	5	Class 1	Complete	Present
2.	MIID-C2	Blood	*K. pneumoniae*	ON506905	*bla*_NDM-1,_ *bla*_OXA-23_	*qnrS*	1.5, 15.2	2	Class 1	Truncated	Present
3.	MIID-C3	Blood	*K. pneumoniae*	ON506906	*bla*_NDM-1,_ *bla*_OXA-23,_ *bla*_OXA-48_	*qnrS*	1.5, 2.5, 2.9, 5.8, 15.2	5	Class 1	Complete	Present
4.	MIID-C4	Blood	*K. pneumoniae*	ON506907	*bla*_NDM-1,_ *bla*_OXA-23,_ *bla*_OXA-48_	*qnrS*	1.5, 2.5, 2.9, 5.8, 15.2	5	Class 1	Complete	Present
5.	MIID-C5	Urine	*K. pneumoniae*	ON506908	*bla*_NDM-1,_ *bla*_OXA-23,_ *bla*_OXA-48_	*qnrS*	1.5, 2.5, 2.9, 5.8, 15.0, 15.2	6	Class 1	Complete	Present
6.	MIID-C6	Urine	*K. pneumoniae*	ON506909	*bla*_NDM-1,_ *bla*_OXA-23,_ *bla*_OXA-48_	*qnrS*	2.5, 4.6, 5.8, 15.5, 18.1	5	Class 1	Complete	Present
7.	MIID-C7	Urine	*K. pneumoniae*	ON506910	*bla*_NDM-1,_ *bla*_OXA-23,_ *bla*_OXA-48_	*qnrS*	1.5, 2.9, 5.8, 15.2, 16.9	5	Class 1	Complete	Present
8.	MIID-C8	Blood	*K. pneumoniae*	ON506911	*bla*_NDM-1,_ *bla*_OXA-23,_ *bla*_OXA-48_	*qnrS*	1.5, 2.5, 2.9, 5.8, 15.2, 16.9	6	Class 1	Complete	Present
9.	MIID-C9	Blood	*K. pneumoniae*	ON506912	*bla*_NDM-1,_ *bla*_OXA-48_	*qnrS*	1.5, 2.5, 2.9, 5.8, 10.0, 15.2, 16.9	7	Class 1	Complete	Present
10.	MIID-C11	Endotracheal aspirate	*K. pneumoniae*	**#**	*bla*_OXA-51,_ *bla*_OXA-48_	*qnrS*	1.5, 2.5, 2.9, 5.8, 15.2	5	**ND**	**NP**	**NP**
11.	MIID-C12	Endotracheal aspirate	*K. pneumoniae*	ON506913	*bla*_NDM-1,_ *bla*_OXA-23,_ *bla*_OXA-48_	*qnrS*	1.5, 2.5, 2.9, 5.8, 15.2	5	Class 1	Complete	Present
12.	MIID-C13	Urine	*K. pneumoniae*	ON506914	*bla*_NDM-1,_ *bla*_OXA-48_ *bla*_OXA-51_	*qnrS*	1.5, 2.5, 2.9, 5.8, 15.2	5	Class 1	Complete	Present
13.	MIID-C15	Blood	*K. pneumoniae*	ON755345	*bla*_NDM-1,_ *bla*_OXA-48_ *bla*_OXA-51_	*qnrS*	1.0, 1.5, 2.9, 5.8, 15.2, 16.9, 17.2	7	Class 1	Complete	Present
14.	MIID-C16	Blood	*K. pneumoniae*	ON506915	*bla*_NDM-1,_ *bla*_OXA-23_	*qnrS*	15.0	1	**ND**	Complete	Present
15.	MIID-C17	Blood	*K. pneumoniae*	ON506916	*bla*_NDM-1,_ *bla*_OXA-48_ *bla*_OXA-51_	*qnrS*	1.0, 1.5, 2.9, 5.8, 15.2, 16.9, 17.2	7	**ND**	Complete	Present
16.	MIID-C18	Blood	*K. pneumoniae*	ON506917	*bla*_NDM-1,_ *bla*_OXA-48_ *bla*_OXA-51_	*qnrS*	1.0, 1.5, 2.9, 5.8, 15.2, 16.9, 17.2	7	Class 1	Complete	Present
17.	MIID-C19	Blood	*K. pneumoniae*	ON506918	*bla*_NDM-1,_ *bla*_OXA-48_ *bla*_OXA-51_	*qnrS*	1.0, 1.5, 2.9, 5.8, 15.2, 16.9, 17.2	7	Class 1	Truncated	Present
18.	MIID-C20	Blood	*K. pneumoniae*	ON493161	*bla*_NDM-1,_ *bla*_OXA-48_ *bla*_OXA-51_	*qnrS*	1.0, 1.5, 2.9, 5.8, 15.2, 16.9, 17.2	7	Class 1	Complete	Present
19.	MIID-C21	Blood	*K. pneumoniae*	ON493162	*bla*_NDM-1,_ *bla*_OXA-48_ *bla*_OXA-51_	*qnrS*	1.0, 1.5, 2.9, 5.8, 15.2, 16.9, 17.2	7	Class 1	Complete	Present
20.	MIID-C22	Blood	*K. pneumoniae*	ON493163	*bla*_NDM-1,_ *bla*_OXA-48_ *bla*_OXA-51_	*qnrS*	1.0, 1.5, 2.9, 5.8, 15.2, 16.9, 17.2	7	Class 1	Complete	Present
21.	MIID-C23	Blood	*K. pneumoniae*	*	*bla*_NDM-1,_ *bla*_OXA-48_	*qnrS*	15.0, 17.2	2	Class 1	Complete	Present
22.	MIID-C24	Blood	*K. pneumoniae*	*	*bla*_NDM-1,_ *bla*_OXA-48_ *bla*_OXA-51_	*qnrS*	2.9, 5.8, 6.7, 17.2	4	Class 1	Truncated	Present
23.	MIID-C25	Blood	*K. pneumoniae*	ON755346	*bla*_NDM-1,_ *bla*_OXA-48_ *, bla*_OXA-51_	*qnrS*	1.5, 2.9, 5.8, 15.2	4	Class 1	Complete	Present
24.	MIID-C26	Blood	*K. pneumoniae*	*	*bla*_NDM-1,_ *bla*_OXA-48,_	*qnrS*	5.8, 15.2	2	Class 1	Complete	Present

*NDM not detected in this isolate, **#**NDM sequence not submitted to GenBank Database.

ND, Not Detected; NP, Not performed.

### Genetic environment of the *bla*_NDM_

**Figure 3:**
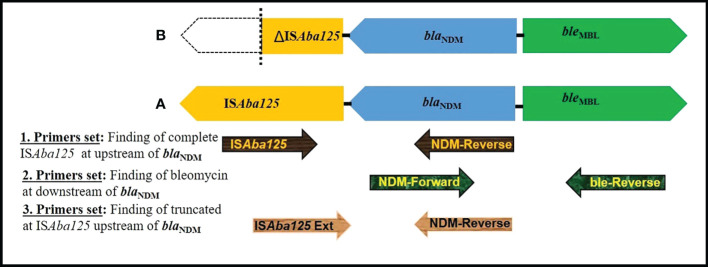
Schematic representation of genetic elements surrounding *bla*NDM. **(A)** In MIID-C1, MIID-C3, MIID-C4, MIID-C5, MIID-C6, MIID-C7, MIID-C8, MIID-C9, MIID-C12, MIID-C13, MIID-C15, MIID-C16, MIID-C17, MIID-C18, MIID-C20, MIID-C21, MIID-C22, MIID-C23, MIID-C25 and MIID-C26 complete element of IS*Aba125* at upstream and bleomycin gene at downstream to *bla*NDM was found. **(B)** (MIID-C2, MIID-C19 and MIID-C24, truncated IS*Aba125* at upstream and bleomycin gene downstream to *bla*NDM was found. Arrow indicates the position of primer (used of primers as mentioned in **Supplementary Table S1**).

In all 23 NDM-1 expressing CRKP isolates, the bleomycin resistance gene (*ble*_MBL_) was identified downstream of the *bla*_NDM-1_ gene by PCR-based genetic environment study of the blaNDM gene. The complete IS*Aba125* sequence was found upstream of *bla*_NDM-1_ in twenty isolates (MIID-C1, MIID-C3, MIID-C4, MIID-C5, MIID-C6, MIID-C7, MIID-C8, MIID-C9, MIID-C12, MIID-C13, MIID-C15, MIID-C16, MIID-C17, MIID-C18, MIID-C20, MIID-C21, MIID-C22, MIID-C23, MIID-C25 and MIID-C26). Furthermore, three *bla*_NDM-1_ carrying *Klebsiella pneumoniae* isolates (MIID-C2, MIID-C19 and MIID-C24) had truncated IS*Aba125*, upstream of *bla*_NDM-1_ as shown in **Table 2** and **Figure 3**.

### Integron analysis

All CRKP clinical isolates carry class 1 integron except three isolates (MIID-C11, MIID-C16 and MIID-C17). The class 2 and class 3 integrons were not found in any CRKP isolates as shown in **Table 2**.

## Discussion

The multidrug-resistant strain of *K. pneumoniae*, a common nosocomial pathogen that regularly causes difficult-to-treat infections globally, is a serious public health concern (Cao et al., 2014). Hospital patients experience higher rates of morbidity and mortality because of rapid CRKP spread, which is the primary cause of treatment failure (Al-Zahrani and Alsiri, 2018). CRKP has also been reported in most of the Gulf Cooperation Council countries (Zowawi et al., 2013). The results of the current CRKP isolates are worrying. All the isolates were multidrug resistant and molecular analysis revealed double or triple carbapenemase gene combinations (NDM-1, OXA-48, OXA-51, OXA-23) with co-existence of qnrS gene. All these isolates carried one to seven number of plasmids varying in size from 1.0 to 18.1 kb. The genetic environment of the NDM-1 carrying isolates revealed majority with downstream of *ble*_MBL_ while three isolates had truncated IS*Aba125*, upstream of *bla*_NDM-1_.

These isolates were highly resistant to most of the commonly used antibiotics including ceftolozane/tazobactam but least resistance to ceftazidime/avibactam and tigecycline. In the United Arab Emirates, ceftolozane-tazobactam and ceftazidime-avibactam were reported as effective alternatives for treating bacteria that produce ESBL and carbapenemase enzymes (Alatoom et al., 2017). Despite being effective against many gram-negative infections, ceftolozane/tazobactam does not show clinically significant potency against Enterobacterales that produce carbapenemase or are carbapenem-resistant (Sutherland and Nicolau, 2015). Accordingly, all the isolates in our study were resistant to ceftolozane/tazobactam. This was in agreement with Alatoom et al. from UAE where their isolates positive for both NDM-1 and OXA-48 showed 100% resistance to ceftolozane/tazobactam (Alatoom et al., 2017). Similarly, Sader et al. also found that all CRKPs were ceftolozane/tazobactam resistant (Sader et al., 2021). Another study from China also reported high resistance of CRKP to ceftolozane/tazobactam (98.1%) (Yin et al., 2019). Contrary to the current study, a study from the United States (Sutherland and Nicolau, 2017) reported 94% ceftolozane/tazobactam susceptibility which was comparable to the 93% previously reported by Farrell and colleagues (Farrell et al., 2014).

Avibactam is a broad-spectrum non-β-lactam β-lactamase inhibitor that inhibits a variety of serine β-lactamases (Bush, 2015). The addition of avibactam to ceftazidime improves ceftazidime effectiveness against common gram-negative bacteria, including the majority of those that produce β-lactamase enzymes and are resistant to carbapenem drugs. In the present study ceftazidime-avibactam had better activity against all the CRKP isolates whereas a study from UAE reported 45% susceptibility to ceftazidime-avibactam (Alatoom et al., 2017). Higher susceptibility rates were also reported by Flamm et al. (Flamm et al., 2014) and Lagace-Wiens et al. (Lagacé-Wiens et al., 2014). In a different trial, Mutter et al. observed good activity of ceftazidime/avibactam against carbapenem-resistant Enterobacterales without carbapenemase synthesis. Ceftazidime-avibactam is also reported to exhibit strong *in vitro* action against Enterobacterales that produce KPC enzymes and may be potent additions to the arsenal of antimicrobial drugs already in use (Kazmierczak et al., 2016). Hence this drug combination can be used as a valuable option for empirical therapy. This also further underscores the requirement for regional studies to assess the impact of novel medications on regional MDR isolates because the prevalence and types of carbapenemase vary among geographical locations (Alatoom et al., 2017).

Since tigecycline and colistin are the drugs of last resort used to treat CRKP infections, the emergence of CRKP strains that also exhibit resistance to these drugs has become a significant clinical challenge (Zhang et al., 2018). Globally, incidences of colistin-resistant CRKP have been recorded as colistin use has increased. A therapeutic issue brought on by the advent of colistin-resistant in CRKP poses a risk of sending patients and physicians back to the “pre-antibiotic period” (Rojas et al., 2017). The isolates tested for colistin and tigecycline resistance in this study demonstrated that 75% were resistant to colistin and 44% showed intermediate susceptibility to tigecycline. While Saeed et al. from Bahrain found that 0.06% of CRE isolates had combination resistance to both colistin and tigecycline (Saeed et al., 2019). A study from Saudi Arabia reported 43.4% colistin resistance among ICU isolates (Al Mayahi et al., 2019). An article by Paris et al. mentions the presence of 590 colistin-resistant *K. pneumoniae* isolates from six Middle East countries, including Saudi Arabia (24), Kuwait (5), United Arab Emirates (31), Iran (86), Turkey (438), Lebanon (3), and Israel (3) between 2013 and 2018, none reported from Bahrain (Aris et al., 2020). In a multicenter observational cohort of hospitalized patients with CRKP in US hospitals, Rojas et al. noted a 13% colistin resistance rate (Rojas et al., 2017). Colistin-resistant CRKP was the subject of a sustained outbreak in Brazil, according to a study, in which 83.9% were from the same cluster and 67.6% hadn’t used polymyxin, indicating the likelihood of cross-transmission of colistin-resistant CRKP isolates (Rocha et al., 2022). According to Sharma et al., all *K. pneumoniae* from NICU and 94.4% from ICU were colistin-resistant CRKP (Sharma et al., 2022). *K. pneumoniae* clinical isolates in the SENTRY Antimicrobial Surveillance Program in 2014 and 2015 showed 4.4% resistant to colistin (Castanheira et al., 2016). Although colistin is effective in treating infections brought on by CRKP, colistin resistance is known to be induced during colistin treatment and can be brought on by mutations and genetic changes in chromosomal genes (Berglund, 2019).

In Africa-Middle East countries, *K. pneumoniae* is reported to show *in vitro* tigecycline susceptibility rates of 96.8% (Renteria et al., 2014). The present study showed 44% intermediate susceptibility to tigecycline whereas a study in Lebanon reported 3% tigecycline-resistant and 16% intermediate findings in *K. pneumoniae* (Araj and Ibrahim, 2008), and research by Park et al, found that CRKP isolates exhibited a 37.8% tigecycline resistance rate (Park et al., 2020). Another study from Egypt stated 10.9% and 36.1% of CRKP isolates were colistin- and tigecycline-resistant, respectively (Gandor et al., 2022). In South Korea, it was revealed that CRKP had a 14.5% tigecycline resistance rate (Jeong et al., 2016). In the United States, multicenter research found that CRKP isolates had an 18.0% tigecycline resistance rate.

The class B β-lactamase NDM-1 has recently raised significant concerns. Additionally, numerous studies have documented the discovery of enterobacterial isolates that produce NDM-1 in various regions of the world (Poirel et al., 2011a). Our study isolates 95.8% (n=23) also were NDM-1 producers along with other carbapenemases (OXA-23, OXA-48, and OXA-51). None of our isolates carried KPC gene. Whereas a study from Saudi Arabia reported 80.9% of isolates with triple resistance genes KPC/NDM-1/OXA-48 while 19.04% carried double resistance genes (KPC/OXA-48) or (NDM-1/OXA-48) (Khan et al., 2019). A study in Turkey reported 38.9% and 81.05% of CRKP isolates as NDM-1 and OXA-48 producers (Genç et al., 2022). Another study from Tehran found 11.5% of isolates as NDM producers along with other beta-lactamases (Sharahi et al., 2021). According to a study from Egypt, 56.2%, and 41.0% of the CRKP isolates showed the presence of blaNDM, and blaOXA-48 respectively.

Beyond sporadic isolates seen in the USA, Denmark, and India, double carbapenemase producers, particularly OXA and NDM co-producing *K. pneumoniae*, are on the rise (Balm et al., 2013; Doi et al., 2014). Additionally, *K. pneumoniae* isolates from Saudi hospitals and numerous other countries in the Arabian Peninsula have both been found to be OXA-48 and NDM positive (Jamal et al., 2016; Al-Agamy et al., 2018).This study also found the co-existence (87.5% (n=21) of *bla*_NDM-1_ and *bla*_OXA-48_ among CRKP isolates. According to a study from the Arabian Peninsula, UAE had 8.9% of CRE isolates that were NDM-1 and OXA-48 co-producers, and other nations in the region reported 1.9% in the Kingdom of Saudi Arabia, 1.6% in Kuwait, and 5.4% in Oman respectively (Sonnevend et al., 2015) which is comparatively low as compared to the present study. A study from Morocco also reported 34% of *K. pneumoniae* as NDM-1 and OXA-48 co-producers (Loqman et al., 2021). Even though three OXA types viz. OXA-23, OXA-48, and OXA-51 were found in our cohort, none of the isolates had simultaneous presence of all three OXA types. However, two OXA types were present together (OXA-23 + OXA-48 & OXA-48 + OXA-51), the more common combination was (OXA-48 + OXA-51, 45.8%).

Among the study isolates, 45.8% and 41.6% were positive for OXA-51, and OXA-23 respectively which is very high as compared to El-Badawy et al. from Egypt, where they noticed 5.3% and 10.5% of the CRKP were capable of producing OXA-23 and OXA-51 like β-lactamases (El-Badawy et al., 2020). In contrast, OXA-23-like and OXA-51-like enzymes were not found in CRKP isolates by Cetinkol et al. (Cetinkol et al., 2016). There is a dearth of information in the literature about NDM-1 and OXA-51, or OXA-23 co-producers in *K. pneumoniae.*

Carbapenems and fluoroquinolones are routinely used to treat severe infections caused by MDR *K. pneumoniae*. The limited treatment options for these strains are caused by the co-existence of plasmid-mediated quinolone resistance (PMQR) determinants and carbapenemases (El-Feky et al., 2017). In the present study, carbapenemases and PMQR co-existed in 100% of our isolates. Whereas a study from Saudi Arabia reported 57% of isolates with co-existence of carbapenemases and qnrS genes (Al-Agamy et al., 2018). However, a study from Egypt reported co-existence of carbapenemases and PMQR genes in 79% of their isolates (El-Feky et al., 2017). A study from China reported that 100% of their carbapenemase-producing *K. pneumoniae* harbored PMQR genes wherein *qnrS* was present in 56% of the isolates (Liu et al., 2014). As commonly observed for carbapenemase producers, all the study strains showed multidrug resistance. All NDM and OXA carbapenemases were highly associated with other resistance genes such as *qnrS* and various mobile elements including class 1 integron, which is comparable with the findings of Liu et al. (Liu et al., 2014) and Huang et al. (Huang et al., 2012).

The transferrable nature of antibiotic resistance is its most worrisome aspect. Multiple mechanisms exist for how plasmids can influence antibiotic resistance (Dong et al., 2018). In this study, we found that majority of the isolates carried five plasmids and every isolate has plasmids ranging in molecular weight from 1 to 18.1 kb. Accordingly, research by Dong et al. found that each isolate included five plasmids, and each measuring 178,177.5, 99.7, 11.9, and 5.6 kb in size (Dong et al., 2018). Another study from the Neonatal intensive care unit of an Indian hospital reported that the 66 kb, 38 kb, and 6 kb plasmid sizes were identified in carbapenem-resistant *K. pneumoniae* clinical isolates (Orole and Hadi, 2020). The presence of many plasmids in these isolates may serve as a potential source for the spread of highly resistant genes to other bacteria and humans, which poses a risk to the commonly used antibiotics to treat infections.

Detection of class 1, 2, and 3 integrons in CRKP isolates in this study showed the majority carrying class 1 integron. The significant association between the presence of class 1 integrons and the occurrence of MDR among *Klebsiella pneumonia*e were previously reported (Li et al., 2013; Malek et al., 2015). Additionally, the fact that the majority of the isolates had the entire ISAba125 sequence upstream of blaNDM-1 (**Figure 3**) suggests that this element may be crucial in the horizontal gene transfer of the blaNDM among members of the Enterobacteriaceae family of bacteria (Poirel et al., 2011b). Moreover, in the current study *ble*_MBL_ was found at its downstream in all *bla*_NDM-1_ producing CRKP isolates. The high rate of association of the *ble*_MBL_ and *bla*_NDM-1_ genes suggests that they might have mobilized together from a common progenitor, which many thought to protect *bla*_NDM_ (Dortet et al., 2012). These results conform with previous reports that clarified the horizontal transfer of plasmids in CRKP isolates (Dortet et al., 2012).

A few limitations apply to this study. The size of the study isolates included was relatively small and this may not be an accurate representation of all CRKP isolates at SMC, Bahrain. Hence a longer-term, further-extended multi-center study is required. In addition, the analysis of resistance determinants by molecular methods was limited to CRKP isolates and did not include the details of ESBL and other antibiotic resistance mechanisms. However, to the best of the authors’ knowledge, this is the first report thoroughly describing the susceptibility profiles, antibiotic-resistant genes and the genetic environment of CRKP isolates in this region.

## Conclusion

Despite the small sample size, the results are alarming. The resistance rate to most antibiotics is very high in our region, including colistin and tigecycline, and the genetic environment of CRKP is complex with the carriage of multiple resistance markers and multiple plasmids. However, without active surveillance, it is impossible to be convinced that these patterns indicate widespread persistence. Resistance to ceftazidime/avibactam is uncommon and hence can be used as a valuable option for empirical therapy. Molecular data on resistance markers and the genetic environment of CRKP is lacking from this geographical region; this would be the first report addressing the subject matter. Our study emphasizes the significance of surveillance programs and strict infection control strategies backed by potent molecular epidemiological tools in clinical settings to curb the emergence and spread of such isolates.

## Data availability statement

The datasets presented in this study can be found in online repositories. The names of the repository/repositories and accession number(s) can be found below: https://www.ncbi.nlm.nih.gov/genbank/, GenBank nucleotide database: ON506904, ON506905, ON506906, ON506907, ON506908, ON506909, ON506910, ON506911, ON506912, ON506913, ON506914, ON506915, ON506916, ON506917, ON506918, ON493161, ON493162, ON493163, ON755345 and ON755346.

## Author contributions

MS conceived the project proposal. NS provided clinical CRKP isolates, identified them and performed antibiotic susceptibility on automated systems. NA, MHS and AAM performed further experiments including molecular experimentation. MS, FD, NS, KB, and KT evaluated the data and provided expertise and feedback. NA, RJ and MS wrote the preliminary draft of the manuscript. All authors contributed to the article and approved the submitted version.

## Funding

Internal funding for the research grant (G06/AGU-11/19) from the Arabian Gulf University.

## Acknowledgments

All authors acknowledge and thank AGU for providing research grant. Dr. Nayeem Ahmad acknowledge and gratefully thank AGU for providing Postdoc Research Fellowship. The authors also acknowledge Genoscreen Lab, France for providing sequence facility support. The authors would like to thank Dr. Amer Almarabheh, PhD, for assistance with statistical analysis. The part of this work was presented as poster presentation at the 70th Annual Conference of the Canadian Society of Microbiologists, University of Guelph, Guelph, Ontario, Canada (June 20- 23, 2022).

## Conflict of interest

The authors declare that the research was conducted in the absence of any commercial or financial relationships that could be construed as a potential conflict of interest.

## Publisher’s note

All claims expressed in this article are solely those of the authors and do not necessarily represent those of their affiliated organizations, or those of the publisher, the editors and the reviewers. Any product that may be evaluated in this article, or claim that may be made by its manufacturer, is not guaranteed or endorsed by the publisher.
