# Supplementary material for: Clinical carbapenem-resistant Klebsiella pneumoniae isolates simultaneously harboring bla NDM-1, bla OXA types and qnrS genes from the Kingdom of Bahrain: Resistance profile and genetic environment
Source: Front Cell Infect Microbiol. 2022 Oct 11;12:1033305. doi: 10.3389/fcimb.2022.1033305 (PMC9592905; doi:10.3389/fcimb.2022.1033305)
Supplement: Supplementary file 1 [file Table_1.docx]

**Table-S1: List of primers used in this study**

| **Gene** | **Primer Sequence (5’-3’)** |
| --- | --- |
| OXA-23 | Forward-ATGAATAAATATTTTACTTG |
|  | Reverse-TTAAATAATATTCAGCTGTT |
| OXA-51 | Forward-TAATGCTTTGATCGGCCTTG |
|  | Reverse-TGGATTGCACTTCATCTTGG |
| OXA-48 | Forward-TTGGTGGCATCGATTATCGG |
|  | Reverse-GAGCACTTCTTTTGTGATGGC |
| NDM | Forward-GGTTTGGCGATCTGGTTTTC |
|  | Reverse-CGGAATGGCTCATCACGATC |
| KPC | Forward-ATGTCACTGTATCGCCGTCT |
|  | Reverse-TTACTGCCCGTTGACGCCCA |
| *IntI-I* | Forward-TCTCGGGTAACATCAAGG |
|  | Reverse-AGGAGATCCGAAGACCTC |
| *IntI-II* | Forward-TTATTGCTGGGATTAGGC |
|  | Reverse-ACGGCTACCCTCTGTTATC |
| *IntI-III* | Forward-AGTGGGTGGCGAATGAGTG |
|  | Reverse-TGTTCTTGTATCGGCAGGTG |
| Bleo | Reverse-GGCGATGACAGCATCATCCG |
| ISAba125A | TGTATATTTCTGTGACCCAC |
| ISAba125ext | ACACCATTAGAGAAATTTGC |
| *qnrA* | Forward- CAGCAAGAGGATTTCTCACG |
|  | Reverse- AATCCGGCAGCACTATTACTC |
| *qnrB* | Forward-GGCTGTCAGTTCTATGATCG |
|  | Reverse-GAGCAACGATGCCTGGTAG |
| *qnrS* | Forward-GCAAGTTCATTGAACAGGGT |
|  | Reverse-TCTAAACCGTCGAGTTCGGCG |
| MCR-1 | Forward- CGGTCAGTCCGTTTGTTC |
|  | Reverse- CTTGGTCGGTCTGTAGGG |
| MCR-2 | Forward- TGTTGCTTGTGCCGATTGGA |
|  | Reverse- AGATGGTATTGTTGGTTGCTG |
| MCR-3 | Forward- TTGGCACTGTATTTTGCATTT |
|  | Reverse- TTAACGAAATTGGCTGGAACA |
